# Vaccine hesitancy amongst healthcare workers corrodes public vaccination trust

**DOI:** 10.1016/j.jvacx.2023.100309

**Published:** 2023-05-12

**Authors:** Weijia Cai, Zuanjun Su, Canye Li, Zhicong Chen, Jinming Cao, Feng Xu

**Affiliations:** Fengxian Hospital, Southern Medical University, Shanghai 201400, China

Trust is the common denominator for COVID-19 vaccine acceptance [1]. Indeed, patient trust in vaccination is very important. But the question is, where does the patient's trust come from? Obviously, the first trust of health workers is even more crucial. Unfortunately, up to now vaccine hesitancy remains a public health threat, which is not unusual healthcare workers.

Since COVID-19 omicron variant infection is still rampant currently [2], it is extremely urgent to overcome the pandemic through enhance vaccination in a wide population, as COVID-19 vaccines are important to improve immunity against pathogens and diseases and to reduce the incidence of the severe form of the disease. Here we would like to add that the trust of healthcare workers in vaccines is very important to improve the public's willingness to vaccinate and their vaccine hesitancy might corrode public vaccination willing.

According to the World Health Organization identification, vaccine hesitance is the reluctance or refusal to vaccinate despite availability of vaccines. It was one of the 10 threats to global health in 2019 [3]. Vaccine hesitancy has never been a simple public health issue. It has a long history, and is no exception at home and abroad [4]. Over the past three years, many approved COVID-19 vaccines are available and accessible; unfortunately, vaccine hesitancy like a phantom, hinders the public to take the vaccine [5].

The COVID-19 'infodemic' continues to weaken trust in vaccination efforts aiming to bring an end to the pandemic. The epidemic and social crisis brought about by the COVID-19 exaggerated the widespread social anxiety and trust problems, and increased people's suspicion of vaccines in the unique environment of the global pandemic [6]. The vaccine hesitancy exists in public as well as in healthcare workers. In China, the overall prevalence of COVID-19 vaccine hesitancy was 8.40% in about 30,000 participants from 31 Chinese provinces [7]. In Shanghai, a super metropolis in China, the vaccination situation is not optimistic. As of June 16, 2022, the vaccination rate of the elderly aged 60 and above in this city is 63.16%. Unfortunately, the unvaccinated population has a high incidence and mortality of serious consequences. Such a low vaccination rate is far from being able to build a barrier for mass immunization [8]. As for healthcare workers, the proportion of vaccine hesitancy was high and the main reason is fear of side effects [9].

Around the world, vaccine hesitancy among healthcare workers is not uncommon. The prevalence of COVID-19 vaccination hesitancy worldwide in healthcare workers ranged from 4.3 to 72% (average = 22.51% across all studies with 76,471 participants). Most of the studies found concerns about vaccine safety, efficacy, and potential side effects as top reasons for COVID-19 vaccination hesitancy in healthcare workers. Most of the studies also found that individuals who were males, of older age, and doctoral degree holders (i.e., physicians) were more likely to accept COVID-19 vaccines [10]. Fear of possible vaccination side effects and the low level of confidence in vaccine safety and effectiveness are the main predictors of vaccine hesitancy among them [11], [12], [13].

There are various factors – individual, interpersonal, healthcare and societal level factors - affecting on COVID-19 vaccine hesitancy [14]. Healthcare worker recommendations as well as sources or credentials of COVID-19-related information are healthcare and societal level factors. Healthcare workers usually have a key role in reducing the burden of the pandemic, role modeling for preventive behaviors, and helping vaccinate others. In many developing countries, healthcare workers generally play a great role in enhancing public intent toward vaccination. Many documents displayed that it is highly effective for healthcare workers to inspire public vaccine uptake by alleviating people's concerns over the vaccines and addressing vaccine-related misinformation which is mainly from social media [15], [16]. In China, people usually seriously consider the opinions of the healthcare workers. In the current COVID-19 vaccine hesitancy period, the comprehensive and accessible vaccine information from authoritative healthcare workers may facilitate COVID-19 vaccination efforts. Effective, timely and positive communication between healthcare workers and the public/patients could combat misinformation and greatly reduce vaccine hesitancy in the vaccination campaign. Here, it is of importance for healthcare worker to rebuild public trust in China vaccine.

During the vaccine communication, if healthcare workers themselves show a little hesitancy, and do not provide public with correct and the latest evidence-based information on vaccine varieties, public vaccine willingness is not possibly promoted. Only if we can win public trust with strong clinical research data, vaccine hesitancy might be overcome.

## Declaration of Competing Interest

The authors declare that they have no known competing financial interests or personal relationships that could have appeared to influence the work reported in this paper.

## Data Availability

No data was used for the research described in the article.
